# Association of Pre-miR-146a rs2910164 Polymorphism with Papillary Thyroid Cancer

**DOI:** 10.1155/2015/802562

**Published:** 2015-11-18

**Authors:** Xin Zhang, Yulu Gu, Xiaoli Liu, Yaqin Yu, Jieping Shi, Qiong Yu, Hui Sun, Joseph Sam Kanu, Siyan Zhan, Yawen Liu

**Affiliations:** ^1^Department of Epidemiology and Biostatistics, School of Public Health, Jilin University, Changchun 130021, China; ^2^Department of Pharmacy, The First Hospital of Jilin University, Changchun 130021, China; ^3^Jilin Provincial Key Laboratory of Molecular Epidemiology, School of Public Health, Jilin University, Changchun 130021, China; ^4^Jilin Provincial Key Laboratory of Surgical Translational Medicine, Department of Thyroid and Parathyroid Surgery, China-Japan Union Hospital, Jilin University, Changchun 130033, China; ^5^Department of Epidemiology and Biostatistics, School of Public Health, Peking University Health Science Centre, Beijing 100191, China

## Abstract

The incidence rate of papillary thyroid cancer (PTC) has increased over the past decades, but the pathogenesis remains unclear. rs2910164, located in pre-miR-146a, has been studied in PTCs with different ethnicity, but the results were inconsistent. Here we evaluate the association between rs2910164 polymorphism and PTC and investigate the effect of this polymorphism on patients' clinicopathological characteristics. 1238 PTC patients and 1275 controls, all Han population, from Northern China, were included in our study. rs2910164 was genotyped using Matrix-Assisted Laser Desorption/Ionization Time of Flight Mass Spectrometry (MALDI-TOF-MS). Analysis of inheritance model was performed using the SNPStats program. Strength of association was assessed by odds ratio (OR) and 95% confidence interval (CI). Overall, no statistical difference in rs2910164 genotype distribution and allelic frequencies between cases and controls was found, and patients with different genotypes had similar clinicopathological characteristics in terms of stage, location, concurrent of benign thyroid tumor, and thyroiditis, while, as the number of G alleles increased, proportion of patients aged ≥45 years and those without metastasis increased (*P*_trend_ < 0.001 and *P*_trend_ = 0.003, resp.). However, no association remained significant after Bonferroni correction under any model of inheritance. Our results suggest no association between rs2910164 polymorphism with PTC and patients' clinicopathological characteristics.

## 1. Introduction

Thyroid cancer is the most common endocrine malignancy with an incidence rate that has doubled over the past ten years and ranked fifth in 2014 (6%) from a previous eighth position (3%) as a cause of all new cancer cases in females [1, 2]. Papillary thyroid cancer (PTC) accounts for about 80% of all histological types of thyroid cancer, but its pathogenesis remains unclear though extensively studied in recent years. The incidence rate of PTC between genders is distinctly different and its inherited genetic predisposition in first-degree relatives is also notable [3–5]. Concerning the genetic factor in the occurrence and development of PTC, a variety of studies, including association studies, genome wide association studies (GWAS), pedigree studies, and meta-analyses, have been carried out [6–9]. Although several single nucleotide polymorphisms (SNPs) of different genes related to PTC have been identified, the results are inconsistent and need further investigation. At present, the well-known mutations that are believed to be associated with PTC include BRAF, RET, RAS, and NTRK1 [10].

MicroRNAs (miRs) are noncoding RNAs that are transcribed from endogenous DNA molecules. The first miR was identified in 1993 and more than one thousand miRs have been discovered in human genome by now [11]. Most miRs span about 22 nucleotides and negatively regulate their target mRNAs in the posttranscriptional processes [12]. The formation of mature miR involves three steps. Firstly, endogenous DNAs are transcribed to form the primary miR products (pri-miRs) with the help of RNA polymerase. Secondly, pri-miRs are cut to form about 70 nucleotides, double-stranded hairpin structures (pre-miRs) by the RNA endonuclease. Finally, the pre-miRs are transported to cytoplasm and cut by Dicer with the leading strand used to form mature miRs (about 22 nucleotides) and the passenger strand degraded. Mature miRs bind to their target mRNAs in the 3′ untranslated regions (3′ UTR) by the so-called seed region, 2–8 nucleotides [13, 14]. miRs regulate diverse cellular processes, including development, differentiation, proliferation, apoptosis, and stress response [15]. miRs downregulate the expression of target mRNAs through translational inhibition, mRNA cleavage, and mRNA decay and can act as tumor suppressors and oncogenes in tumorigenesis [16, 17].

Pre-miR-146a C/G polymorphism, designated rs2910164, is encoded on chromosome 5q33 and located in the precursor stem region, +60 relative to the first nucleotide of pre-miR-146a, opposite to the mature miR-146a sequence [13]. It is the only miR sequence SNP studied in PTC and has been demonstrated to be associated with a variety of cancers, such as lung cancer, prostate cancer, gastric cancer, and oral cancer [18–21]. Moreover, a meta-analysis on this SNP and cancer predisposition suggested that gender and smoking status played a role in the effect [22]. Studies on its contribution to PTC have been performed in different races, including European and Asian populations, but the results were inconsistent [6, 13, 23, 24]. Jazdzewski et al. first demonstrated that rs2910164 played a role in genetic predisposition to PTC through regulation of miR expression [13], but later studies carried out with either European or Asian population found no evidence of association between this polymorphism and risk of PTC, although Wei et al. revealed a possible role of rs2910164 in transforming benign thyroid tumor to PTC [6, 23, 24]. Hence, we carried out the present study to further verify whether any association exists between rs2910164 polymorphism and PTC.

## 2. Materials and Methods

### 2.1. Study Population and Ethics Statement

With a case-control study design, 1238 PTC patients and 1275 healthy controls were involved in our study. All PTC patients were pathologically diagnosed and received surgical treatment at the China-Japan Union Hospital of Jilin University in Changchun, Jilin province, China, from January 2010 to December 2014. The histological type of the PTCs was classical PTC, not the follicular variant PTC (fvPTC). The controls consisted of two groups: 360 participants recruited from the First Bethune Hospital of Jilin University in Changchun, Jilin province, China, for a routine health examination, as previously published [25], and 915 from a large-scale community-based cross-sectional survey of chronic disease and risk factors among adults in Jilin province in July 2012. Individuals with metabolic disease, thyroid disease, or malignancy were excluded from the control group. For 360 controls from the First Bethune Hospital of Jilin University, the diseases mentioned above were excluded by routine examinations, while, for controls from the survey, these diseases were self-reported. Subjects of both groups were Northern Chinese Han population. The study was approved by the ethics committee of the School of Public Health, Jilin University, and written informed consent was obtained from all individual participants in the study.

### 2.2. DNA Extraction and Genotyping

For DNA extraction, about 5 mL peripheral venous blood was collected from each subject and stored at −20°C in nonanticoagulant, plexiglass tubes with the ClotBlood DNA kit (Cwbio, Beijing, China) used for genomic DNA extraction and ultraviolet spectrophotometer (Beckman, USA) used for measuring the concentration and purity of the extracted DNA.

For SNP genotyping, we used the Assay Designer 3.1 to design the primers of polymerase chain reaction (PCR): 5′-ACGTTGGATGCCACGATGACAGAGATATCC-3′ (forward) and 5′-ACGTTGGATGGAACTGAATTCCATGGGTTG-3′ (reverse). PCR was performed as previously published [26]. The genotyping of rs2910164 was done by MassARRAY system (Sequenom, San Diego, CA, USA) with the technology of Matrix-Assisted Laser Desorption/Ionization Time of Flight Mass Spectrometry (MALDI-TOF-MS).

### 2.3. Statistical Analysis

Continuous variables were presented with mean and standard deviation and tested using independent samples *t*-test between groups. Chi-square test was used to evaluate the departure from Hardy-Weinberg equilibrium (HWE) in controls and to compare the difference between cases/controls or subgroups of categorical variables. Analysis of inheritance model was performed using the online SNPStats program (http://bioinfo.iconcologia.net/SNPStats) [27]. Strength of risk for developing PTC was assessed by odds ratio (OR) and its 95% confidence intervals (CI). Bonferroni correction was performed to reduce type I error in multiple testing. All the analyses were performed using SPSS 16.0 unless otherwise specified, and two-sided test with *P* value less than 0.05 was considered statistically significant.

## 3. Results

### 3.1. Characteristics of the Study Subjects

Our study included 1238 PTC cases and 1275 healthy controls. The mean ages of cases and controls were 43.43 and 43.38 years, respectively, and the female-to-male rate ratio was more than 2.5 in both groups. Distributions of age and gender were similar between the two groups (*P* > 0.05), and our study subjects were balanced. Among the cases, 90.6% and 86.4% had no prior self or family history of thyroid disease, respectively, and 11.2% had ever smoked. A majority of patients were at an early stage of disease and 33.6% had lymph node metastasis. Cancer tissues were mostly located at unilateral thyroid (70.5%) and more than one-fourth were bilateral. About half (50.9%) of all patients were diagnosed with thyroid microcarcinoma (a subtype of thyroid cancer with tumor diameter less than 1.0 cm). Besides, 611 (51.5%) patients had benign thyroid cancer(s) simultaneously, including 582 nodular goiter, 35 simple goiter, and 74 adenoma. Among them, 167 patients (39.3%) had their benign tumor on the same side of thyroid gland as the PTC. Moreover, 108 patients had thyroiditis, accounting for 8.7%. The characteristics of the study subjects are shown in Table 1.

### 3.2. Association between rs2910164 Polymorphism and PTC Risk

HWE test indicated that the genotype distribution in control group was consistent with that expected under the HWE (*P* = 0.628). No statistically significant difference in rs2910164 genotype distribution and allelic frequencies was observed between cases and controls (Table 2). To investigate the possible role of rs2910164 in PTC, we carried out the inheritance model analysis between two groups adjusted by age and gender using the SNPStats online procedure. As shown in Table 3, we found no evidence of a significant association under any model of inheritance (*P* > 0.05 for codominant, dominant, recessive, overdominant, and log-additive models).

### 3.3. Association between rs2910164 Polymorphism and Clinicopathological Characteristics

Table 4 shows the comparison of clinicopathological characteristics among patients with different rs2910164 genotypes. Patients with different genotypes had similar clinicopathological characteristics in stage, location, tumor size, concurrent of benign thyroid tumor, and thyroiditis (Table 4). However, with the increasing number of G alleles, proportion of patients aged ≥45 years and those without metastasis increased (*P*_trend_ < 0.001 and *P*_trend_ = 0.003, resp.). To further estimate the effect of rs2910164 polymorphism on PTC clinicopathological characteristics, we performed the inheritance model analysis adjusted by age and gender. In general, codominant, dominant, recessive, overdominant, and log-additive models were conducted to compare the clinical characteristics among patients with different genotypes (Table 5). For stage of disease, patients with later stage (stages III–IV) were compared with those with earlier stage (stages 0–II); and for tumor location on thyroid gland, patients with cancer located bilaterally were compared with those located unilaterally. Specially, cancers that were restricted to the thyroid isthmus were treated as unilateral location. As shown in Table 5, no evidence of association was found under any model of inheritance between rs2910164 polymorphism and stage, metastasis, location, concurrent of benign thyroid tumor, or thyroiditis (*P* > 0.05), while, compared with CC homozygotes, patients with CG genotype were more likely to have smaller tumor size under the codominant model (OR = 0.73, 95% CI 0.56–0.96, *P* = 0.022), and similar result was found for CG/GG carriers under the dominant model (OR = 0.74, 95%  CI 0.57–0.96, *P* = 0.025). However, no association remained statistically significant after Bonferroni correction. Therefore, we speculated that no association between rs2910164 polymorphism and PTC or clinicopathological characteristics existed.

## 4. Discussion

Scientific research on gene polymorphisms involved in carcinogenesis and cancer progression has been a hot area of interest for several decades and the advent of the next generation sequencing technology and GWAS is accelerating the pace of development in this area. Many studies have revealed the importance of miRs in various biological processes, including their roles in several cancers (e.g., thyroid cancer), and provided us with useful information [28–30].

Pre-miR-146a was first found in mouse and several studies have shown that it plays an important role in tumorigenesis and cancer metastasis [31–34]. The G>C polymorphism caused C:U mismatch in the stem region from G:U pair, leading to decreased expression of pre-miR-146a and mature miR-146a [13]. In our current study, we demonstrated no significant difference in rs2910164 genotype distribution and allelic frequencies between cases and controls (Table 2). The genotype frequencies in our study were 30.2%, 51.0%, and 18.7% for CC, CG, and GG, respectively, which were similar to the results of a study by Wei and colleagues carried out in a Han Chinese population (CC, 36.4%; CG, 45.4%; GG, 18.2%) [24], but discrepant with the report by Jazdzewski et al. on Caucasians (CC, 6.1%; CG, 35.5%; GG, 58.4%) [13]. Wei and colleagues' study revealed no significant association in genotype distribution and allele frequency between PTCs, benign thyroid tumor patients, and controls, while, in Jazdzewski and team's study, genotype distributions were significantly different between cases and controls (*P* < 0.001), and heterozygote CG was found to exert a significant influence on PTC occurrence. Thus, we speculate that ethnic diversity may have an influence on the effect of rs2910164 on PTC. Two other studies on participants in Europe (Italia and UK) had similar genotype distribution with that of Jazdzewski et al. but found no association between rs2910164 and PTC [6, 23]. One of these studies had relatively small sample size [6], which might have affected the results. This implies that not only ethnic diversity but also other factors such as lifestyles in different countries (which play an important role in a variety of cancers) may have effects on such an association.

In some retrospective case-control studies carried out on Chinese Han ethnic subjects, rs2910164 has been found to be associated with the risk of several kinds of cancers. Zhou et al. found that the GG genotype of rs2910164 was significantly associated with an increased risk of gastric cancer, which was more notable in the pooled analysis when another follow-up replication study was added, and the association was more significant in younger individuals than the older ones [35]. This finding was similar to that by Zeng et al., who also demonstrated a higher risk of gastric cancer in subjects of 58 years or younger carrying GC/GG genotype compared with homozygotes of CC [36]. Guo et al. evaluated the influence of rs2910164 polymorphism on esophageal squamous cell carcinoma (ESCC) and observed that GG homozygote as a risk factor functioned in ESCC susceptibility and clinical stage [37]. Moreover, rs2910164 GG genotype was demonstrated to be related to occurrence of prostate cancer and cervical cancer in Chinese males and females [38, 39]. In our study, no model of inheritance presented significant association between rs2910164 genotype and PTC. The inconsistency between the above studies and ours may be due to the distinct roles of rs2910164 in different types of cancer.

It should be mentioned that several meta-analyses tried to explain the association between rs2910164 and cancer risk but got no significant results. Two studies reviewed genetic association studies on common microRNA polymorphisms and hepatocellular carcinoma susceptibility, but no significant association was found for rs2910164 [40, 41]. Moreover, other meta-analyses evaluated the effect of rs2910164 polymorphism on cancer risk but no evidence of relationship between this SNP and the overall cancer risk was found [42–44]. However, in subgroup analysis, strong association with overall cancer risk was found in Caucasian populations in a study by K. Srivastava and A. Srivastava [42]. Wang et al. observed a significant association of cancer risk in subgroup analysis for PTC, primary liver cancer, cervical cancer, as well as Caucasian population and small sample size studies [43]. A study by Ma et al. found that C allele or CC genotype was a protective factor for bladder cancer, prostate cancer, cervical cancer, and colorectal cancer, but a risk factor for PTC and squamous cell carcinoma of the head and neck. These findings further support our explanation that the effect of rs2910164 polymorphism on cancer risk differs in different ethnicity and cancer type, and sample size may also have an influence on any association reported.

Age, as a well-recognized prognostic determinant in well-differentiated thyroid cancer (WDTC), has been adopted in many thyroid cancer predictive models and prognostic scoring systems [45–48]. According to the American Joint Cancer Committee/Union Internationale Contre le Cancer Tumor Nodes Metastasis (AJCC/UICC TNM) staging system, patients aged ≥45 years are an independent risk factor for prognosis of WDTC [49]. Age may exert its influence in thyroid cancer prognosis indirectly through other factors, such as radioactive iodine responsiveness, thyroid stimulating hormone (TSH) stimulation, luteinizing hormone (LH), and follicle-stimulating hormone (FSH) homology, genetic variation, and overall mortality [50]. Therefore, we speculate that one or more of the above mechanisms might involve age and rs2910164 polymorphism simultaneously, and thus the effect of rs2910164 on PTC clinical characteristics might be influenced by age.

Our study demonstrated no association between rs2910164 polymorphism and risk of PTC, as well as its clinicopathological characteristics, in a Chinese Han population. Our results should be interpreted with caution for the ethnicity restriction and relatively small sample size. Furthermore, a considerable number of controls who self-reported no metabolic disease, thyroid disease, or malignancy were included in the study, which might be a potential for bias in the study. However, it is necessary to conduct that further studies with larger sample size are needed to validate our results, and studies with more detailed data estimating the interaction between rs2910164 and environmental factors may provide a better understanding of the role of rs2910164 in PTC development. Nevertheless, our study provides evidence for further studies on different ethnicity and larger sample size in this field.

## Figures and Tables

**Table 1:** Characteristics of the study subjects.

Variables	Cases (*n* = 1238)	Controls (*n* = 1275)	*t*/*χ* ^2^	*P*
Age	43.43 ± 9.204	43.38 ± 9.311	−0.123	0.902
Gender				
Male	321 (25.9)	339 (26.6)	0.132	0.716
Female	916 (74.1)	936 (73.4)		
History of thyroid disease				
Yes	114 (9.4)			
No	1093 (90.6)			
Family history				
Yes	160 (13.6)			
No	1017 (86.4)			
Smoking				
Nonsmokers	1066 (88.8)			
Smokers	119 (9.9)			
Ex-smokers	15 (1.2)			
Stage				
0-I	597 (81.7)			
II	36 (4.9)			
III	35 (4.8)			
IV	63 (8.6)			
Metastasis (pN+)				
Yes	399 (33.6)			
No	788 (66.4)			
Location				
Unilateral	787 (70.5)			
Bilateral	311 (27.9)			
Isthmus	18 (1.6)			
Diameter				
≤1 cm	598 (50.9)			
>1 cm	578 (49.1)			
Benign tumor				
Yes	625 (51.5)			
No	588 (48.5)			
PTC/benign				
Same side	176 (29.3)			
Opposite sides	425 (70.7)			
Thyroiditis				
Yes	106 (8.7)			
No	1107 (91.3)			

**Table 2:** Genotype and allele frequencies of the cases and controls.

rs2910164	Cases	Controls	*χ* ^2^	*P*
Genotype				
CC	386 (32.0)	392 (31.5)	0.079	0.961
CG	601 (49.8)	619 (49.8)		
GG	221 (18.3)	232 (18.7)		
Allele				
C	1373 (56.8)	1403 (56.4)	0.077	0.781
G	1043 (43.2)	1083 (43.6)		

**Table 3:** Association between rs2910164 genotypes and PTC under different models of inheritance (adjusted by age and gender).

Model	Genotype	Cases	Controls	OR (95% CI)	*P*
Codominant	CC	383 (31.8)	392 (31.5)	1.00	
	CG	600 (49.8)	619 (49.8)	0.97 (0.80–1.18)	0.85
	GG	221 (18.4)	232 (18.7)	0.94 (0.74–1.21)	0.71

Dominant	CC	383 (31.8)	392 (31.5)	1.00	
	CG/GG	821 (68.2)	851 (68.5)	0.96 (0.80–1.16)	0.69

Recessive	CC/CG	983 (81.6)	1011 (81.3)	1.00	
	GG	221 (18.4)	232 (18.7)	0.96 (0.77–1.20)	0.72

Overdominant	CC/GG	604 (50.2)	624 (50.2)	1.00	
	CG	600 (49.8)	619 (49.8)	0.99 (0.84–1.18)	0.93

Log-additive	—	—	—	0.97 (0.86–1.10)	0.64

**Table 4:** The clinical characteristics of the PTC cases by rs2910164 genotypes.

Variables	CC	CG	GG	*χ* ^2^	*P*	*P*_trend_
Gender						
Male	99 (25.6)	152 (25.3)	60 (27.1)	0.283	0.868	0.74
Female	287 (74.4)	448 (74.7)	161 (72.9)			
Age						
<45	268 (70.0)	323 (53.7)	72 (32.6)	79.974	**<0.001**	**<0.001**
≥45	115 (30.0)	278 (46.3)	149 (67.4)			
History of thyroid disease						
Yes	34 (9.0)	61 (10.5)	14 (6.5)	3.109	0.211	0.471
No	345 (91.0)	521 (89.5)	203 (93.5)			
Family history						
Yes	49 (13.2)	86 (15.2)	23 (10.9)	2.512	0.285	0.634
No	323 (86.8)	481 (84.8)	188 (89.1)			
Smoking						
Nonsmokers	337 (90.6)	515 (88.3)	189 (87.9)	7.009^a^	0.182	0.1
Smokers	33 (8.9)	62 (10.6)	20 (9.3)			
Ex-smokers	2 (0.5)	6 (1.0)	6 (2.8)			
Stage						
0-I	189 (82.9)	287 (82.7)	107 (78.1)	5.971	0.426	0.521
II	8 (3.5)	17 (4.9)	11 (8.0)			
III	14 (6.1)	13 (3.7)	6 (4.4)			
IV	17 (7.5)	30 (8.6)	13 (9.5)			
Metastasis (pN+)						
Yes	145 (39.0)	179 (31.3)	59 (27.7)	9.453	**0.009**	**0.003**
No	227 (61.0)	393 (68.7)	154 (72.3)			
Location						
Unilateral	244 (70.1)	377 (69.8)	149 (74.9)	4.958	0.292	0.217
Bilateral	95 (27.3)	157 (29.1)	47 (23.6)			
Isthmus	9 (2.6)	6 (1.1)	3 (1.5)			
Diameter						
≤1 cm	168 (46.2)	307 (54.1)	113 (52.6)	5.831	0.054	0.07
>1 cm	196 (53.8)	260 (45.9)	102 (47.4)			
Benign tumor						
Yes	183 (48.3)	309 (52.8)	119 (54.3)	2.673	0.263	0.12
No	196 (51.7)	276 (47.2)	100 (45.7)			
PTC/benign						
Same side	50 (28.4)	81 (27.3)	36 (31.6)	0.751	0.687	0.632
Opposite sides	126 (71.6)	216 (72.7)	78 (68.4)			
Thyroiditis						
Yes	30 (7.9)	61 (10.4)	15 (6.8)	3.247	0.197	0.923
No	349 (92.1)	524 (89.6)	204 (93.2)			

^a^Fisher's exact test.

*P* < 0.05 in bold.

**Table 5:** Association of rs2910164 and clinical characteristics under different models of inheritance with OR (95% CI) (adjusted by age and gender).

Characteristics	Codominant 1	Codominant 2	Dominant	Recessive	Overdominant	Log-additive
Stage (III-IV versus 0–II)	0.65 (0.39–1.09)	0.75 (0.37–1.55)	0.67 (0.41–1.10)	1.02 (0.55–1.91)	0.73 (0.46–1.13)	0.82 (0.57–1.18)
Metastasis (yes versus no)	0.84 (0.63–1.12)	0.86 (0.58–1.28)	0.85 (0.64–1.11)	0.97 (0.68–1.37)	0.88 (0.69–1.14)	0.91 (0.75–1.11)
Location (bilateral versus unilateral)	1.20 (0.88–1.63)	0.99 (0.64–1.51)	1.15 (0.85–1.55)	0.87 (0.60–1.26)	1.20 (0.92–1.57)	1.02 (0.83–1.26)
Diameter (>1 cm versus ≤1 cm)	**0.73 (0.56–0.96)**	0.79 (0.55–1.13)	**0.74 (0.57–0.96)**	0.97 (0.71–1.32)	0.80 (0.63–1.01)	0.86 (0.72–1.03)
Benign tumor (yes versus no)	1.12 (0.86–1.46)	1.10 (0.77–1.56)	1.12 (0.87–1.44)	1.02 (0.75–1.38)	1.08 (0.86–1.36)	1.06 (0.89–1.26)
Thyroiditis (yes versus no)	1.47 (0.92–2.36)	1.04 (0.53–2.03)	1.37 (0.87–2.18)	0.80 (0.44–1.43)	1.45 (0.97–2.19)	1.08 (0.80–1.47)

Codominant 1: CG versus CC; codominant 2: GG versus CC; dominant: CG/GG versus CC; recessive: GG versus CC/CG; overdominant: CG versus CC/GG.

Significant OR (95% CI) in bold.
